# Identification of differentially expressed lncRNAs and candidate ceRNA networks across Mastitis stages in Sahiwal cattle

**DOI:** 10.1371/journal.pone.0357796

**Published:** 2026-09-15

**Authors:** Bharati Pandey, Chetna Tyagi

**Affiliations:** 1 ICAR-National Dairy Research Institute (NDRI), Karnal, Haryana, India; 2 Department of Biotechnology and Microbiology, Faculty of Science and Informatics, University of Szeged, Szeged, Hungary; Chung-Ang University, KOREA, REPUBLIC OF

## Abstract

Along with being a significant disease in dairy cattle which incurs economic loss, mastitis also affects a wide range of parameters that involve milk yield and quality. However, the role of long non-coding RNAs (lncRNAs) in the regulation of mastitis in distinct stages of the disease in Sahiwal cows is yet to be understood. This study characterised lncRNA expression profiles and competing endogenous RNA (ceRNA) networks across healthy, subclinical, and clinical mastitis stages using RNA-Seq data from milk somatic cells. Five biologically relevant ceRNA modules were identified: Five ceRNA modules were identified that are biologically relevant: MSTRG.21615.2, is predicted to sponge bta-miR-760-3p and bta-miR-212, and involved in co-regulation of OAT and SIRT2, leading to dysregulation of urea-cycle and NAD ⁺ -metabolism; MSTRG.19791.1-bta-miR-2382-5p, which activates an extracellular antioxidant network (SOD3, CAT, GPX family, PARK7); MSTRG.1150.15-bta-miR-92b, which regulates the PTEN/PI3K-AKT signalling axis; MSTRG.19363.1-bta-miR-1296, involved in the nuclear import machinery (KPNA7, RCC1, RANBP1, NUP50), with reduced expression in clinical versus subclinical mastitis; and MSTRG.25768.1-bta-miR-2425-5p, linked to extracellular matrix remodelling via the ADAMTS. The binding prediction of all six lncRNA-miRNA pairs were favourable, with the highest predicted affinity observed for MSTRG.1150.15-bta-miR-92b (−15.4 ± 6.9 kcal/mol) and moderate but biologically relevant binding for MSTRG.19363.1-bta-miR-1296 (−7.8 ± 4.2 kcal/mol). Molecular dynamics over 50 ns revealed that the MSTRG.19363.1-bta-miR-1296 complex was the most stable, exhibiting lowest RMSD and radius of gyration values, suggesting that it may be a viable regulator of nuclear transport during the progression of mastitis. These results shed light on the underlying mechanisms of lncRNA-regulated networks associated with dairy cattle mastitis.

## 1. Introduction

Over the past decade, growing evidence has revealed that the non-coding regions of the genome are just as crucial as protein-coding genes in regulating cellular functions. In addition to messenger RNAs (mRNAs), animal genomes produce a diverse array of non-coding RNA molecules that, although they do not encode proteins, play essential regulatory roles in gene expression and cellular processes. Among these, long non-coding RNAs (lncRNAs), defined as transcripts longer than 200 nucleotides with limited or no protein-coding potential, have emerged as key regulators in various biological systems [1]. Despite their structural resemblance to mRNAs, lncRNAs primarily function as modulators of gene activity, influencing processes such as cell proliferation, differentiation, apoptosis, chromatin modification, epigenetic regulation, transcriptional interference, and nuclear transport [2,3].

Bovine mastitis is an inflammation of the udder tissue in dairy cows, usually caused by physical injury or microbial infection. It is one of the most common and costly diseases in the dairy industry, as it reduces milk yield and quality [4,5]. On average, mastitis causes an economic loss of about $147 per cow per year, mainly due to reduced milk production and the need to cull affected animals. This represents about 11–18% of the total profit margin per cow annually [6]. In fact, around 70% of these losses come from damage to mammary tissue that lowers milk production [7]. Mastitis is classified into three main types based on the severity of inflammation: clinical, subclinical, and chronic. Clinical mastitis is easily recognized, as the udder becomes red, swollen, and painful, and the milk appears watery or contains flakes and clots; in severe cases, it can be fatal [8]. Subclinical mastitis shows no visible signs but leads to reduced milk yield and increased somatic cell count (SCC), often causing greater economic losses. Chronic mastitis persists for months, with recurring episodes of inflammation at irregular intervals [9].

In dairy cattle, lncRNAs have been implicated in the regulation of milk production, mammary gland development, and disease resistance. Transcriptomic analyses have identified several lncRNAs associated with milk quality traits, including XLOC_059976 in Chinese Holstein cows, which has been proposed as a regulator of milk protein synthesis [10]. Functional studies further demonstrated that lncRNAs participate in diverse biological processes within the mammary gland. For example, lncRNA H19 promotes bovine mammary epithelial cell (MAC-T) proliferation, β-casein expression, tight junction integrity, and inflammatory responses through NF-κB signaling, whereas LncRNA XIST negatively regulates inflammation by suppressing NF-κB activation and NLRP3 inflammasome formation [11,12]. Moreover, integrated transcriptomic analyses of mastitis-affected cattle have identified complex mRNA-miRNA-lncRNA regulatory networks involving key inflammatory regulators, highlighting the central role of lncRNAs in mastitis pathogenesis [13]. Novel lncRNAs such as lncRNA-TUB were shown to regulate epithelial cell growth, β-casein secretion, inflammation, and bacterial adhesion [14]. Transcriptomics analysis of LPS-stimulated MAC-T cells identified 2,257 lncRNAs (2,047 novel), with 112 differentially expressed lncRNAs implicated in NF-κB, MAPK, PI3K-Akt, mTOR, and Notch signaling [15]. Additionally, LncRNA CA12-AS1 forms a pro-inflammatory axis with miR-133a, modulating NF-κB signaling, cytokine secretion, apoptosis, tight junction integrity, and cell proliferation, demonstrating the diverse regulatory roles of lncRNAs in mastitis [16].

Recent transcriptomic investigations have begun to uncover the role of lncRNAs in mastitis. Asselstine et al. (2024) analyzed milk somatic cell transcriptomes and identified 94 differentially expressed lncRNAs associated with bovine mastitis [17]. Functional annotation and QTL analyses revealed that many of these lncRNAs are involved in immune and inflammatory responses, offering valuable insights into breeding strategies aimed at improving mastitis resistance and enhancing milk quality. However, such studies have largely been limited to a single population and do not account for the dynamic progression of the disease, particularly the subclinical stage.

In this context, the present study aims to investigate the transcriptomic landscape of lncRNAs and their associated competing endogenous RNA (ceRNA) networks in Sahiwal cows across healthy, subclinical, and clinical mastitis stages. By integrating RNA sequencing data with computational prediction of lncRNA-miRNA-mRNA interactions, this study provides a comprehensive analysis of lncRNA associated with mastitis progression. This integrative approach not only addresses the existing gap in understanding lncRNA-mediated regulatory networks across different disease stages but also contributes to advancing current knowledge of non-coding RNA regulation in bovine mastitis.

## 2. Materials and methods

### 2.1. Dataset

The RNA-Seq dataset used in this study was retrieved from the NCBI Sequence Read Archive (SRA) under the BioProject accession number PRJNA1214580, which focuses on transcriptomic profiling of milk somatic cells in Sahiwal cows affected by mastitis. The dataset includes samples in triplicate for three groups: clinical mastitis (n = 3), subclinical mastitis (n = 3), and healthy controls (n = 3). RNA sequencing libraries were prepared using the NEBNext Ultra II Directional RNA Library Prep Kit (New England Biolabs) following standard protocols at Genotypic Technology Pvt. Ltd., Bangalore, India. Briefly, 100–500 ng of total RNA was used for mRNA enrichment, followed by fragmentation and priming. First- and second-strand cDNA synthesis was performed, and the resulting double-stranded cDNA was purified using NEBNext sample purification beads. The purified cDNA underwent end repair, adenylation, and ligation with Illumina-compatible adapters. Strand specificity was maintained through second-strand excision using USER enzyme treatment. Adapter-ligated fragments were PCR-amplified (11–14 cycles) to enrich the sequencing libraries, followed by purification using NEBNext beads. Library quality and concentration were assessed using a Qubit fluorometer, and fragment size distribution was evaluated using an Agilent 2200 TapeStation. Sequencing was performed on the Illumina NovaSeq X Plus platform, generating paired-end reads. Data retrieval was carried out using the SRA Toolkit [18], employing prefetch to download  .sra files and fasterq-dump to convert them into paired-end FASTQ files for subsequent quality assessment and analysis.

### 2.2. Read mapping and alignment

Raw paired-end RNA-Seq reads were initially assessed for quality using FastQC (v0.12.1) (https://www.bioinformatics.babraham.ac.uk/projects/fastqc/). Subsequent pre-processing, including adapter removal and quality trimming, was carried out using fastp (v1.3.3) [19] to ensure high-quality reads for downstream analysis. Reads were aligned to the *Bos indicus* NIAB-ARS_B.indTharparkar_mat_pri_1.0 reference genome (GCF_029378745.1) using HISAT2 (v2.2.2) [20], producing SAM files. The alignment files in SAM format were converted to BAM format using SAMtools (v 1.19.2), followed by sorting and indexing. To ensure data quality, all BAM files were verified for integrity, excluding any truncated or corrupted files. Only correctly sorted and indexed BAM files were used for downstream transcript assembly and quantification.

### 2.3. Transcript assembly and quantification

Transcript assembly was performed using StringTie v2.2.1 [21] in reference-guided mode with the corresponding genome annotation (GTF). For each sample, StringTie generated transcript and gene-level expression estimates. Individual transcript assemblies from all samples were then merged into a non-redundant, unified transcriptome annotation, representing the comprehensive transcript landscape across all samples.

### 2.4. Identification of high-confidence long non-coding RNAs (lncRNAs)

To identify high-confidence lncRNAs from the transcriptome, we first selected multi-exon transcripts longer than 200 nucleotides from the merged transcript assembly (merged_annot.annotated.gtf). Only novel transcripts classified under class_code u (unkown), i (intronic), or x (exonic) were considered as candidate lncRNAs. The coding potential of the remaining transcripts was further evaluated using CPC2 (Coding Potential Calculator 2) [22], Coding-Non-Coding Index (CNCI) version 2 (https://github.com/www-bioinfo-org/CNCI) PLEKv2 [23]. These candidate sequences were then aligned against the SwissProt protein database using Diamond BLASTX [24] with an e-value cutoff of 1e-5 and identity >30 to identify potential protein-coding transcripts. Query sequences that produced significant hits in the protein database were considered likely protein-coding and excluded from further analysis. The remaining sequences, which did not show any significant homology to known proteins, were retained as putative non-coding lncRNAs. Finally, to remove residual protein-coding potential, predicted coding sequences identified by TransDecoder v5.7.1 (https://github.com/TransDecoder/TransDecoder) were filtered out. The resulting dataset represents high-confidence lncRNAs, suitable for downstream functional annotation and expression analyses.

### 2.5. Differential expression analysis

Differential expression of lncRNAs was performed by PyDESeq2 [25] (implementation of DESeq2 in Python) using transcript-level count data generated by prepDE.py script. Raw count data and sample metadata were used for pairwise comparisons among healthy, clinical, and subclinical groups. The DESeq2 framework was applied for normalization, dispersion estimation, and differential expression analysis using a negative binomial model. Genes with p-value < 0.05 and absolute log2 fold change ≥ 1 were considered significantly differentially expressed. lncRNA with positive log2 fold change were classified as upregulated, while those with negative log2 fold change were classified as downregulated. The final differentially expressed lncRNAs results were exported as CSV files for downstream analysis. To identify previously annotated bovine lncRNAs, the sequences were aligned against the NONCODE v5 cattle database (NONCODEv5_cow.fa.gz; http://v5.noncode.org/) using BLASTn. LncRNAs with similarity > 80% and *E* < 1e-5 were classified as known lncRNAs. The subcellular localization of the identified lncRNAs was predicted using lncLocator (http://www.csbio.sjtu.edu.cn/bioinf/lncLocator/index.html). Protein-protein interaction (PPI) analysis was performed using the STRING database (v12.0) [26]. No ethical permissions were required for this study.

### 2.6. Target prediction of lncRNAs and ceRNA network construction

Differentially expressed lncRNAs were categorized into upregulated and downregulated groups based on a p-value threshold of <0.05. The corresponding nucleotide sequences of these lncRNAs were retrieved and used for miRNA target prediction using RNAhybrid (v2.1.2; -e −30 -p 0.05) [27] and miRanda (-sc 150 -en −20) [28]. Experimentally validated bovine (*Bos taurus*) miRNA sequences were collected from miRTarBase [29]. The common lncRNA-miRNA interactions identified by both prediction tools were subsequently used for target gene prediction and functional annotation. Functional descriptions of the predicted target genes were obtained from the UniProt database (https://www.uniprot.org/). The identified lncRNA-miRNA-mRNA interactions were then integrated with protein-protein interaction (PPI) information to construct ceRNA-PPI regulatory networks using Py4Cytoscape (v1.13.0) and Cytoscape (v3.10.1).

### 2.7. Three dimensional modelling of lncRNA and miRNA

The secondary structure of differentially expressed lncRNA were generated using RNAfold from the ViennaRNA package [30], which predicts structures with MFE and ensemble properties. Thermodynamic stability was further assessed with RNAeval [31]. For lncRNAs with sequence lengths less than 500 nucleotides, the complete lncRNA sequence was used for secondary structure prediction and subsequent structural analyses. However, for lncRNAs longer than 500 nucleotides, a fragment encompassing the predicted miRNA-binding site along with approximately 100 nucleotides of upstream and downstream flanking sequences was selected for structural modeling. This strategy was adopted due to the sequence length limitations of the currently available online RNA 3D structure prediction tools. The resulting fragment length was typically between 200 and 250 nucleotides, ensuring that the complete interaction region was retained. For tertiary structure prediction, the RNAfold-derived secondary structures and corresponding sequences were submitted to RNAComposer [32] to generate 3D models, which were downloaded in PDB format and visualized in PyMOL 3.1.6.1 [33].

### 2.8. Docking between LncRNA-miRNA

To explore the structural nature of the predicted six lncRNA-miRNA interactions, data-driven molecular docking was carried out on the High Ambiguity Driven Protein–Protein DOCKing (HADDOCK) 2.4 web server [34]. The regions of the docking residues were demarcated as the predicted interacting nucleotide regions by miRanda and RNAhybrid (S1 File). Active and passive residues were automatically assigned by HADDOCK server on the basis of solvent accessibility and spatial proximity to the active residues. The score of HADDOCK, the Z score, the root mean square deviation (RMSD), the van der Waals energy, the electrostatic energy, the desolvation energy, the restraint violation energy and the buried surface area (BSA) were used to rank the resulting docked complexes.

### 2.9. MD simulation of the lncRNA-miRNA

Molecular dynamics (MD) simulation using GROMACS 2024.3 [35] is a powerful approach to study the stability and dynamic behaviour of lncRNA-miRNA complexes after docking. The AMBER99SB-ILDN protein, nucleic AMBER94 force field is usually applied to define atomic interactions. The processed complex was placed at the centre of a cubic simulation box, ensures a minimum distance of 1.2 nm between the protein and the box edges. Appropriate counter ions were then added to neutralize the system. Energy minimization was performed using steepest descent minimization to remove steric clashes and optimize system geometry, and convergence was assessed based on potential energy and maximum force criteria (Fmax < 1000 kJ mol − ¹ nm − ¹). This was followed by equilibration in two phases: first under the constant number, volume, and temperature (NVT ensemble) for 100 ps, during which the system temperature was stabilized at 298 K using the V-rescale thermostat. Subsequently, equilibration was performed under the constant number, pressure, and temperature (NPT ensemble) for 500 ps to stabilize density and pressure, using similar cutoff parameters and long-range electrostatics treatment. In both equilibration phases the Verlet cutoff scheme with grid-based neighbour searching was applied with a 1.2 nm cutoff for van der Waals and electrostatic interactions, while long-range electrostatics were treated using the Particle Mesh Ewald method, initial velocities were generated from a Maxwell-Boltzmann distribution under periodic boundary conditions and bond lengths involving hydrogen atoms were constrained using the LINCS algorithm. After successful equilibration, a 50 ns production molecular dynamics simulation was carried out. The resulting trajectories are analyzed using the inbuilt GROMACS analysis utilities.

## 3. Results

### 3.1. Dataset quality

**Fig 1:**
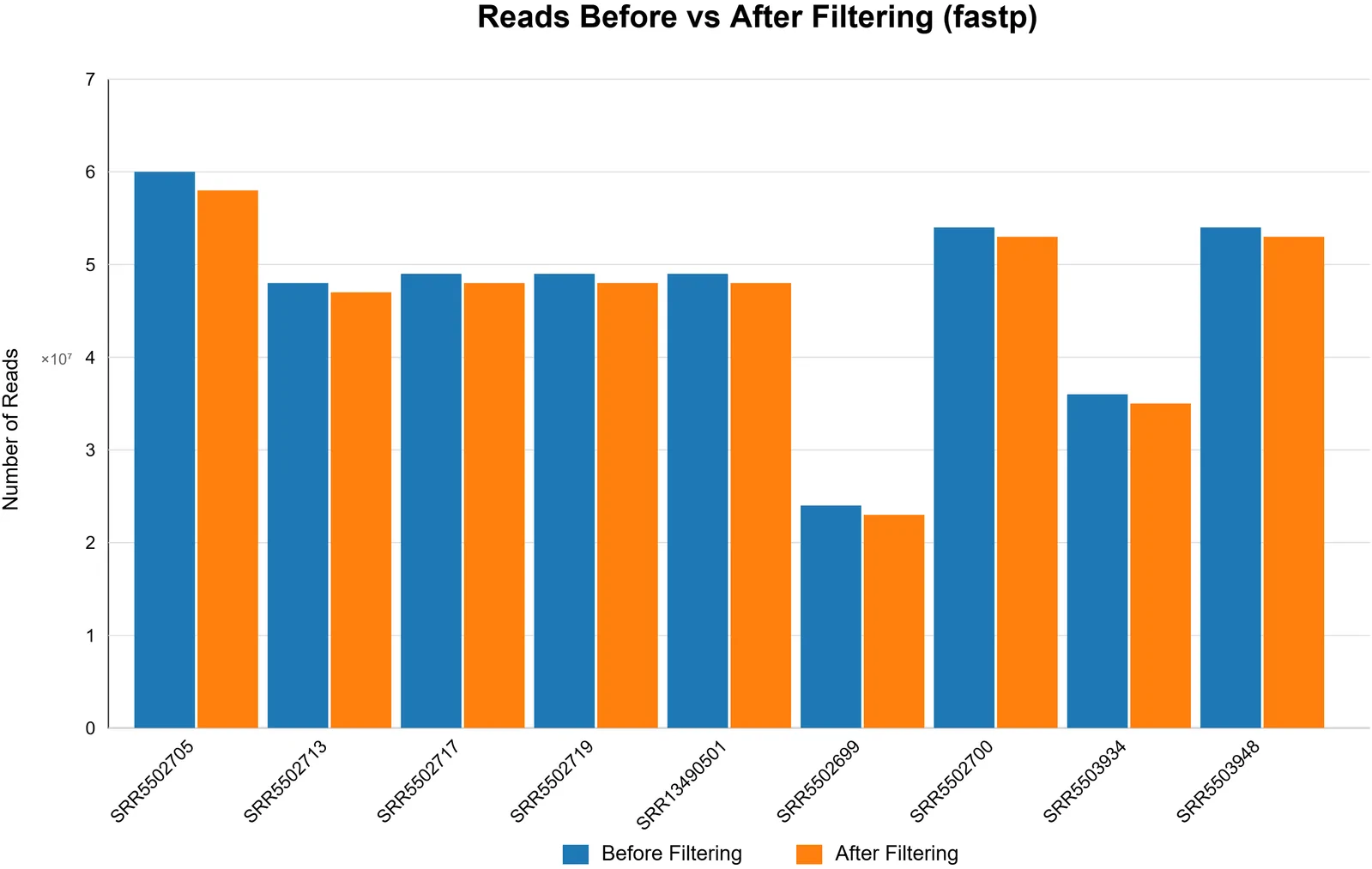
Comparison of read counts before and after quality filtering using fastp.

The sequencing datasets from samples SRR32560793 to SRR32560801 were first evaluated in terms of read quality and alignment efficiency. After processing with fastp, only a minor reduction in read counts was observed between pre- and post-filtering stages, indicating that the raw reads were generally of high quality and required minimal trimming or removal. Samples such as SRR32560797, SRR32560795, SRR32560798, SRR32560800, and SRR32560801 retained nearly all reads after filtering, reflecting excellent sequencing quality, while SRR32560794 and SRR32560799 had relatively fewer total reads overall, which may reduce downstream depth of coverage (Fig 1).

**Fig 2:**
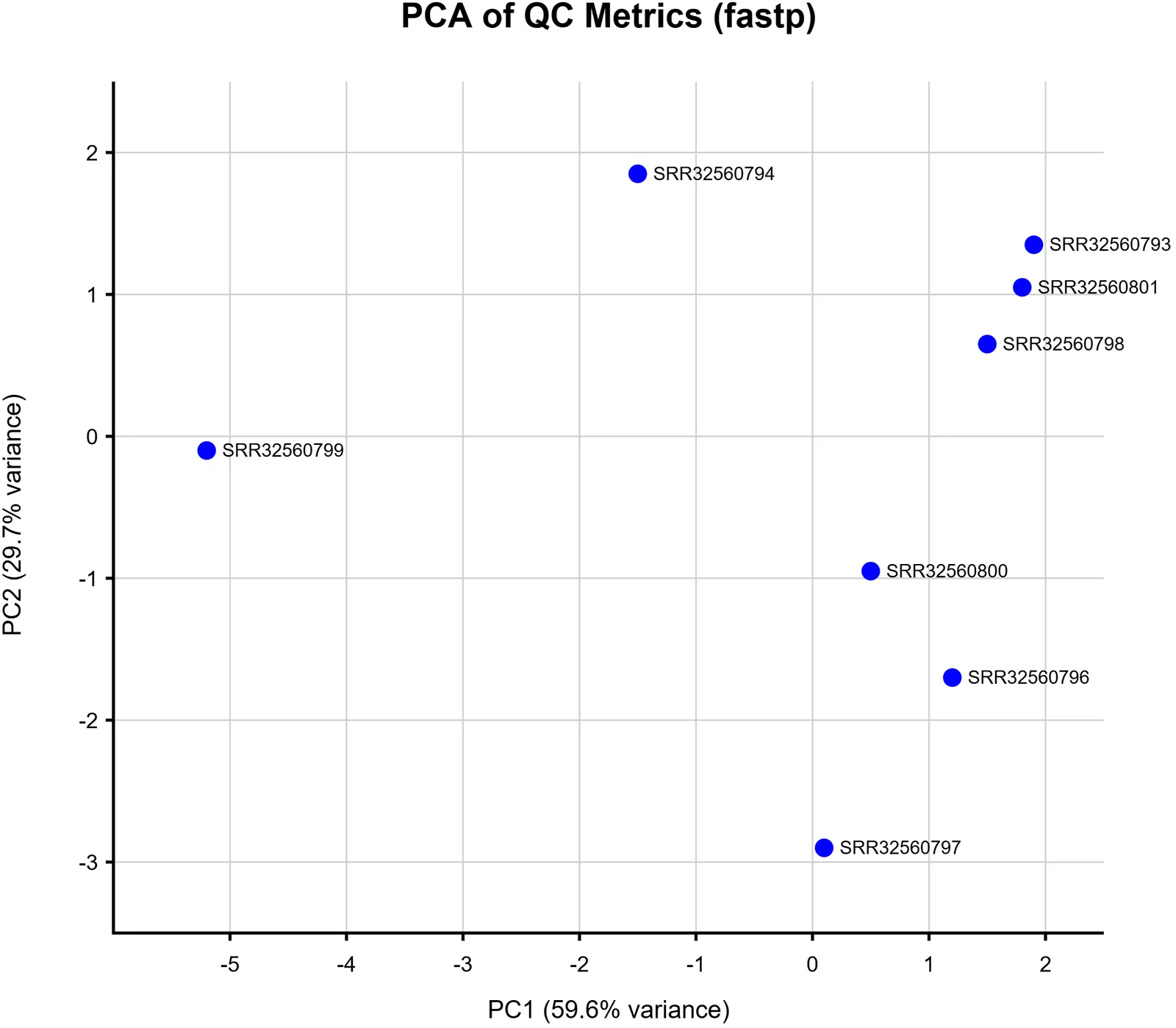
Principal Component Analysis (PCA) of QC metrics generated by Fastp.

The PCA plot of **quality control (**QC) metrics from fastp summarizes multiple quality parameters into two principal components, PC1 (59.6% variance) and PC2 (29.7% variance), together explaining nearly 90% of the variability in the dataset. Most samples, including SRR32560793, SRR32560801, SRR32560798, and SRR32560795, cluster closely on the right side of the plot, indicating consistent sequencing and filtering performance, while SRR32560800 and SRR32560796 show slight separation along PC2, reflecting minor differences QC metrics without indicating outlier behaviour. In contrast, SRR32560794, SRR32560799, and SRR32560797 appear as clear outliers, with SRR32560794 shifted strongly along PC2, SRR32560799 separated along PC1, and SRR32560797 positioned lower on PC2, suggesting distinct deviations likely caused by lower sequencing depth, reduced read quality, or higher filtering losses (Fig 2). These PCA findings align with earlier filtering and mapping results, confirming that most datasets are reliable for downstream analysis, but a few samples with divergent quality metrics require careful consideration.

### 3.2. Alignment with reference genome

**Fig 3:**
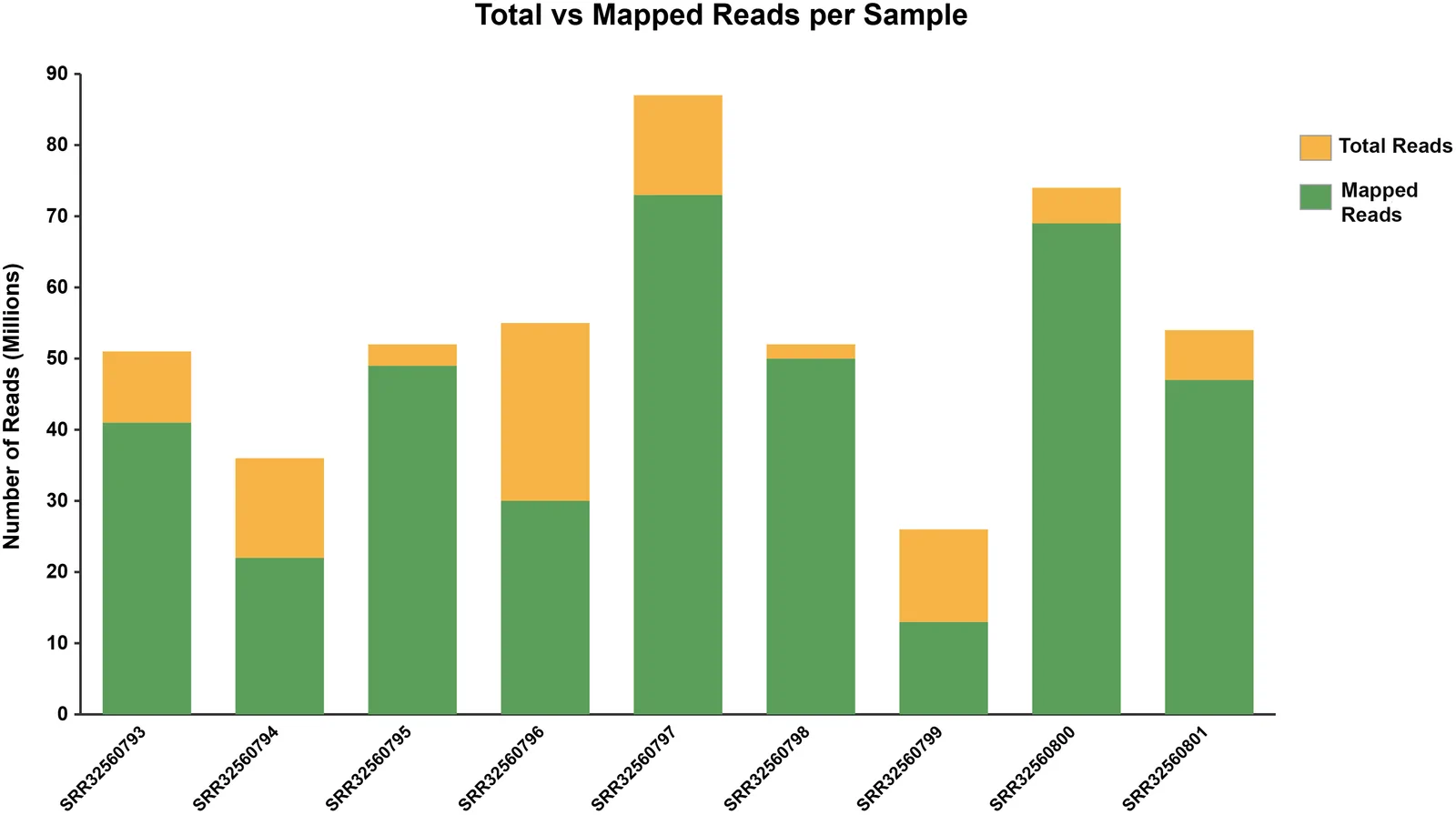
Total vs. mapped reads per sample. Stacked bar plot showing total (orange) and mapped (green) reads for each RNA-Seq sample.

In most samples, the majority of reads successfully mapped to the reference genome, indicating high sequencing and alignment quality. SRR32560797 showed the highest sequencing depth with nearly 87 million total reads, of which around 73 million mapped, followed closely by SRR32560800, which had ~ 75 million total reads and ~70 million mapped reads, both reflecting excellent performance. Samples such as SRR32560795 and SRR32560798 also demonstrated high mapping efficiency, with more than 90% of reads aligning successfully. In contrast, SRR32560794 and SRR32560799 displayed lower sequencing depth and fewer mapped reads, with SRR32560799 being the weakest performer overall (~27 million total reads). These findings indicate that while most datasets are of high quality and suitable for downstream analysis, a few samples with reduced sequencing depth or lower mapping efficiency may limit the robustness of comparative studies (Fig 3).

### 3.3. Identification of lncRNAs

**Fig 4:**
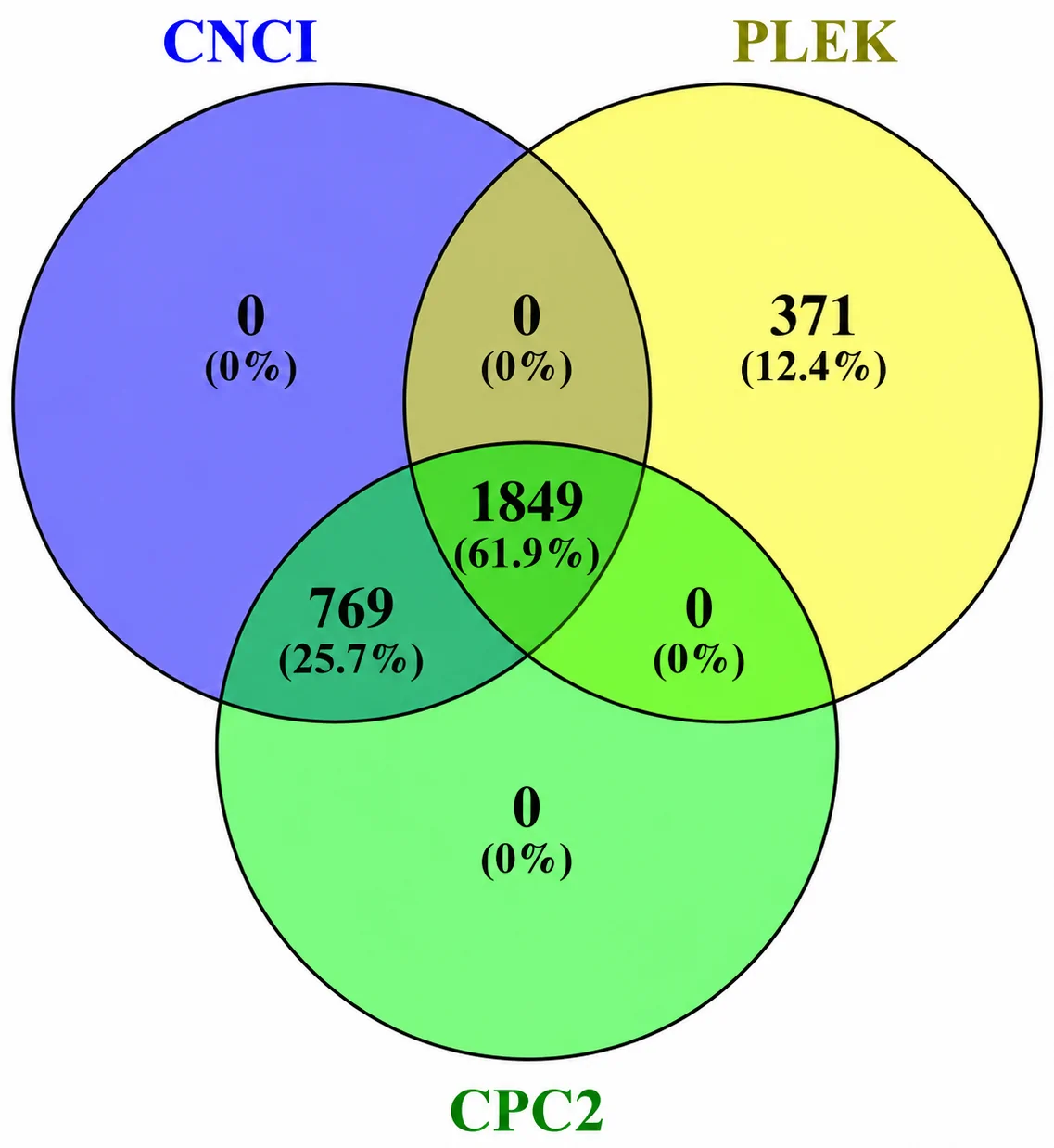
Venn diagram showing the overlap of non-coding transcripts predicted by CNCI, PLEK, and CPC2.

**Fig 5:**
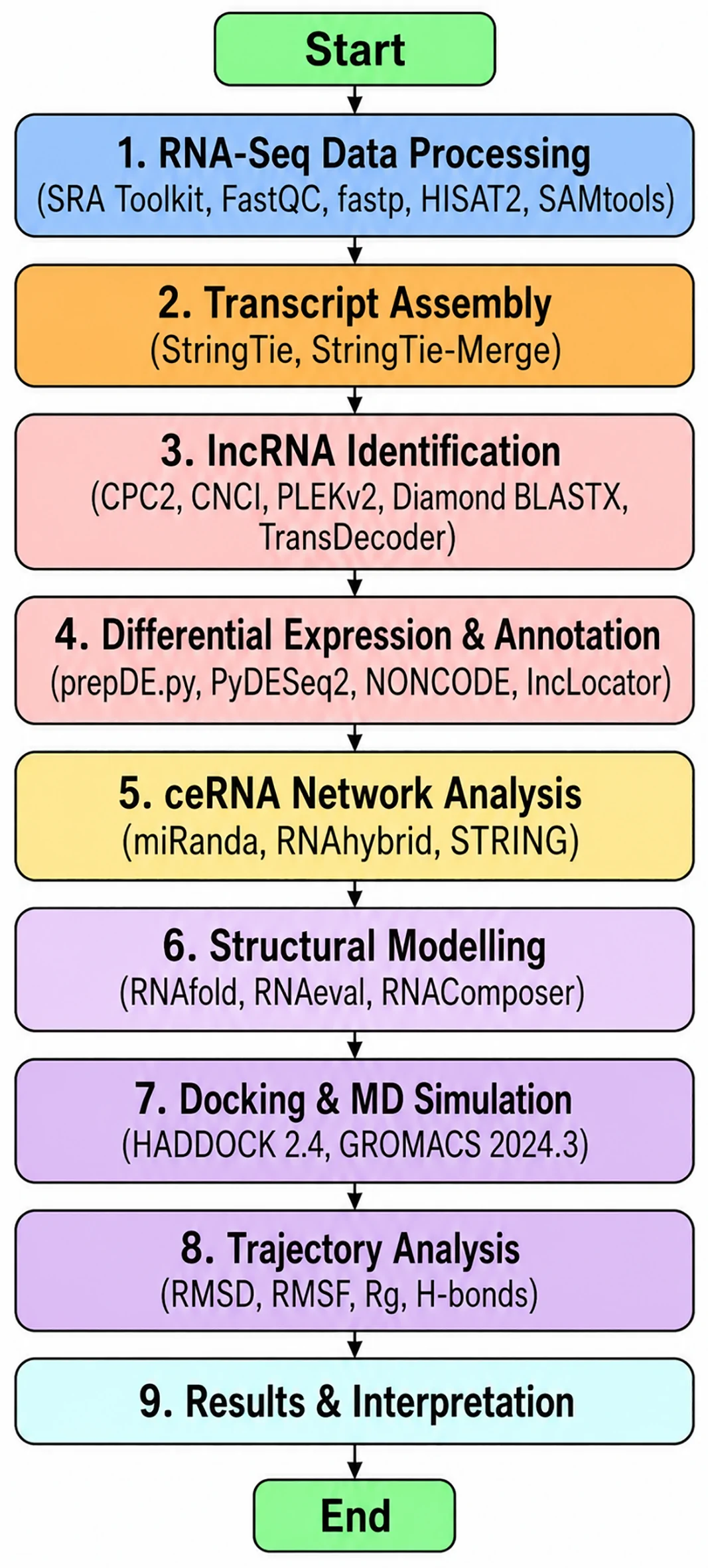
Pipeline for lncRNA identification. Workflow showing sequential steps for identifying high-confidence lncRNAs from RNA-Seq data, including quality control, read alignment, transcript assembly, filtering, and removal of protein-coding transcripts. The flow diagram was generated using Graphviz (https://graphviz.org/).

Among the assembled transcripts, novel transcripts classified under the class codes “u” (unknown intergenic), “i” (intronic), and “x” (exonic overlap on the opposite strand) were considered potential lncRNA candidates. A total of 3,435, 4,607, and 918 transcripts belonged to the “i”, “u”, and “x” categories, respectively. A comprehensive and multi-step filtering pipeline was then applied to identify high-confidence long non-coding RNAs (lncRNAs). Initially, a total of 3613 transcripts were retained after filtering for multi-exonic transcripts (exon number ≥ 2) and transcript length greater than 200 nucleotides, representing the preliminary set of putative lncRNAs. Coding potential of the putative lncRNAs were assessed by three coding-potential prediction tools: CNCI, PLEK, and CPC2. A total of 1,849 transcripts (61.9%) were commonly predicted as non-coding by all three software tools, representing the most reliable set of putative lncRNAs. In addition, 769 transcripts (25.7%) were jointly predicted as non-coding by CNCI and CPC2, while 371 transcripts (12.4%) were uniquely identified by PLEK (Fig 4). The predicted lncRNA candidates were further screened against protein databases using sequence similarity search to remove potential protein-coding transcripts. Transcripts showing significant similarity to known proteins, with sequence identity ≥ 30% and E-value ≤ 1 × 10^−5^, were considered coding-like and excluded from further analysis, leaving 1,428 transcripts for subsequent analysis. To ensure the exclusion of transcripts with significant open reading frames (ORFs), TransDecoder analysis was performed, identifying 757 non-coding transcripts. Finally, by integrating the results from all filtering and coding-potential analyses, a total of 757 transcripts were confidently classified as high-confidence lncRNAs. These represent the most reliable lncRNA candidates, which were subsequently used for downstream structural, functional, and expression analyses (Fig 5).

### 3.4. Differential expression of lncRNA

**Fig 6:**
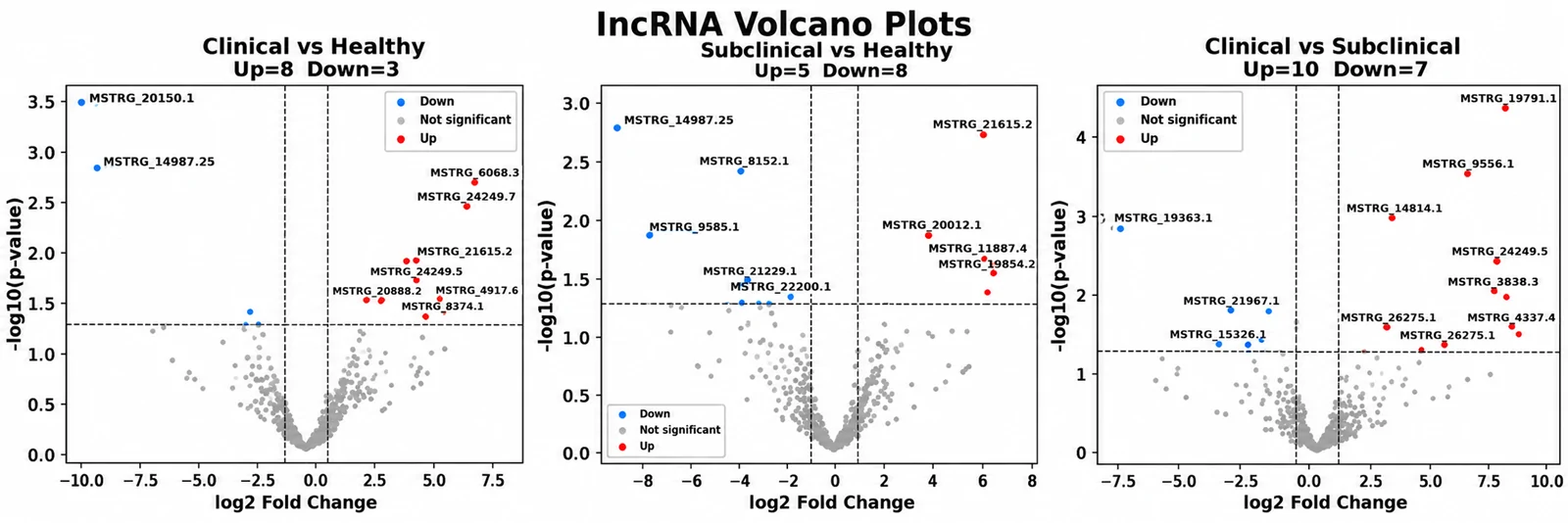
Volcano plots depicting differential gene expression between health status groups. (A) Clinical_vs_Healthy, (B) Subclinical_vs_Healthy, and (C) Clinical_vs_Subclinical. Each point represents an individual gene. Red dots represent upregulated genes, blue dots represent downregulated genes, and grey dots denote non-significant genes. The horizontal dashed line indicates the p-value threshold, and the vertical dashed green lines mark the log2FC cutoff values.

Eleven lncRNAs were found to be significantly dysregulated during the comparison of Clinical and Healthy groups in Sahiwal cattle (Fig 6; Table 1). Of these, eight were found to be upregulated and 3 were downregulated in clinically affected animals. The upregulated transcripts included MSTRG.520.1, MSTRG.3838.2, MSTRG.4917.6, MSTRG.6068.3, MSTRG.8374.1, MSTRG.21615.2, MSTRG.24249.7, and MSTRG.24249.5, with log2FoldChange values ranging from 2.78 to 8.85. Notably, MSTRG.6068.3 exhibited the highest level of upregulation (log2FC = 8.847782, p = 0.001605), followed by MSTRG.24249.7 (log2FC = 8.379593, p = 0.002566). Similarly, MSTRG.21615.2 (log2FC = 6.418643, p = 0.013503) and MSTRG.4917.6 (log2FC = 6.145024, p = 0.027226) also showed marked upregulation. On the other hand, the expression of three lncRNAs, MSTRG.8763.1, MSTRG.14987.25 and MSTRG.20150.1, was significantly downregulated. Among them, MSTRG.20150.1 demonstrated the strongest repression with a log2FoldChange of −9.71796 (p = 0.000305), followed by MSTRG.14987.25 with a log2FoldChange of −8.77464 (p = 0.00127), and MSTRG.8763.1 with a log2FoldChange of −2.53307 (p = 0.043674) (Fig 6; Table 1).

**Table 1:** Significant differentially expressed (DE) lncRNAs in Sahiwal cows with Clinical mastitis in comparison to healthy cows.

Transcript	Log2FoldChange	pvalue	Regulation	Length	Exon_Count	Coding potential by CPC2	Subcellular localization	Chromosome
MSTRG.520.1	2.783801	0.029118	Up	4592	2	0.0718768	Cytosol	Chr 1
MSTRG.3838.2	3.301074	0.02941	Up	2519	5	0.0318245	Ribosome	Chr 3
MSTRG.4917.6	6.145024	0.027226	Up	717	3	0.014664	Cytoplasm	Chr 5
MSTRG.6068.3	8.847782	0.001605	Up	2106	2	0.118962	Cytoplasm	Chr 5
MSTRG.8374.1	5.725796	0.041726	Up	215	2	0.0206381	Exosome	Chr 7
MSTRG.8763.1	−2.53307	0.043674	Down	683	2	0.0380512	Nucleus	Chr 8
MSTRG.14987.25	−8.77464	0.00127	Down	2489	2	0.0528893	Cytosol	Chr 15
MSTRG.20150.1	−9.71796	0.000305	Down	2566	2	0.0968881	Cytosol	Chr 19
MSTRG.21615.2	6.418643	0.013503	Up	917	2	0.114632	Cytoplasm	Chr 22
MSTRG.24249.7	8.379593	0.002566	Up	1253	3	4.99639E-7	Nucleus	Chr 26
MSTRG.24249.5	4.419764	0.015819	Up	1522	4	8.86623E-6	Nucleus	Chr 26

The structural features of the identified lncRNAs were consistent with the characteristics of lncRNAs, with an average length of 1256 bp, ranging from 215 bp to 4592 bp and two to five exons. The longest transcript (4592 bp) was MSTRG.520.1 and the shortest transcript (215 bp) was MSTRG.8374.1. The exon distribution also highlighted the complexity of the transcripts, with the greatest number of exons in MSTRG.3838.2 (five), followed by MSTRG.24249.5 (four) and most other transcripts having two or three. All transcripts exhibited extremely low coding probability scores when analyzed by Coding Potential Analysis (CPC2) further confirming their non-coding status. In particular, the coding potential values for the MSTRG.24249.7 and MSTRG.24249.5 transcripts were extremely low (4.99639E-7 and 8.86623E-6, respectively) and the other transcripts also yielded values well below the protein-coding threshold.

Subcellular localization analysis showed that the identified lncRNAs displayed distict subcellular localization patterns, suggesting functional diversity. Multiple transcripts, such as MSTRG.4917.6, MSTRG.6068.3, and MSTRG.21615.2, were predicted to associate with the cytoplasm, and MSTRG.520.1, MSTRG.14987.25, and MSTRG.20150.1, were associated with the cytosol. When the localization of an RNA is cytoplasmic and/or cytosolic, it is typically involved in post-transcriptional regulatory mechanisms including those such as miRNA sponging, mRNA stability modulation, and ceRNA interactions. Conversely, MSTRG.8763.1, MSTRG.24249.7, and MSTRG.24249.5 were expected to remain in the nucleus, and might be involved in the regulation of transcription, chromatin remodeling or epigenetic control of gene expression. MSTRG.3838.2 was predicted to localize to the ribosome, suggesting a potential role in translational regulatory processes while MSTRG.8374.1 was predicted to localize to the exosome. The differentially expressed lncRNAs were distributed on different chromosomes, such as chromosomes 1, 3, 5, 7, 8, 15, 19, 22 and 26. Chromosome 5 showed the highest enrichment of differentially expressed lncRNAs such as MSTRG.4917.6, MSTRG.6068.3, and MSTRG.8374.1, indicating the potential presence of a lncRNA regulatory cluster on this chromosome. In Sahiwal cattle, this was followed by chromosome 26 where both MSTRG.24249.7 and MSTRG.24249.5 were localized to the nucleus and found to be strongly upregulated, suggesting that chromosome 26 may represent another lncRNA hotspot associated with clinical mastitis in the context of clinical disease in Sahiwal cattle (Fig 6; Table 1).

Differential expression analysis revealed thirteen significantly dysregulated lncRNAs between Subclinical and Healthy groups of Sahiwal cattle (Fig 6; Table 2). Of these, five lncRNAs were found to be upregulated and eight lncRNAs were found to be downregulated, suggesting that there was a general trend towards transcriptional suppression in subclinically affected animals. The upregulated transcripts included MSTRG.11587.4, MSTRG.19854.2, MSTRG.20012.1, MSTRG.20132.1, and MSTRG.21615.2, with MSTRG.21615.2 exhibiting the highest upregulation (log2FC = 7.207271, p = 0.002062), followed by MSTRG.19854.2 (log2FC = 6.905805, p = 0.024803). Among the downregulated transcripts, MSTRG.14987.25 demonstrated the strongest repression (log2FC = −8.83785, p = 0.001785), followed by MSTRG.9585.1 (log2FC = −7.27022, p = 0.013602) and MSTRG.20150.1 (log2FC = −5.82929, p = 0.00114), while MSTRG.2181.1, MSTRG.3051.1, MSTRG.8152.1, MSTRG.12200.1, and MSTRG.21229.1 showed moderate downregulation (Fig 6; Table 2).

**Table 2:** Significant differentially expressed (DE) lncRNAs in Sahiwal cows with Subclinical mastitis in comparison to healthy cows.

Transcript	log2FoldChange	pvalue	regulation	Length	Exon_ Count	Coding potential by CPC2	subcellular localization	Chromosome
MSTRG.11587.4	5.795617	0.021015	Up	2060	2	0.0413046	Cytosol	Chr11
MSTRG.19854.2	6.905805	0.024803	Up	1380	2	0.0311258	Cytoplasm	Chr19
MSTRG.20012.1	4.352484	0.01514	Up	416	2	0.00559583	Cytoplasm	Chr19
MSTRG.20132.1	6.477865	0.040303	Up	347	2	0.0272287	Nucleus	Chr19
MSTRG.21615.2	7.207271	0.002062	Up	917	2	0.114632	Cytoplasm	Chr22
MSTRG.2181.1	−1.68363	0.045516	Down	2009	2	0.0599682	Cytoplasm	Chr2
MSTRG.3051.1	−3.72033	0.049982	Down	282	2	0.0129139	Cytoplasm	Chr3
MSTRG.8152.1	−4.07972	0.003373	Down	285	3	0.0409375	Cytoplasm	Chr7
MSTRG.12200.1	−2.73218	0.034533	Down	385	2	0.0307923	Cytoplasm	Chr11
MSTRG.14987.25	−8.83785	0.001785	Down	2489	2	0.0528893	Cytosol	Chr15
MSTRG.20150.1	−5.82929	0.00114	Down	2566	2	0.0968881	Cytosol	Chr19
MSTRG.21229.1	−3.50037	0.028744	Down	203	2	0.0924648	Cytosol	Chr21
MSTRG.9585.1	−7.27022	0.013602	Down	267	2	0.0472975	Nucleus	Chr9

The lengths of the transcripts ranged from 203 bp (MSTRG.21229.1) to 2566 bp (MSTRG.20150.1) and the number of exons ranged from two to three, typical characteristics of lncRNAs. All transcripts were identified as non-coding by CPC2 based coding potential analysis with values well below the protein-coding threshold. Subcellular localization analysis revealed that most of the transcripts that were cytoplasmic, including MSTRG.19854.2, MSTRG.20012.1, MSTRG.21615.2, MSTRG.2181.1, MSTRG.3051.1, MSTRG.8152.1, and MSTRG.12200.1, may participate in post-transcriptional regulation, such as miRNA sponging and ceRNA interactions. Cytosol-associated were predicted were MSTRG.11587.4, MSTRG.14987.25, MSTRG.20150.1 and MSTRG.21229.1; and nuclear were MSTRG.20132.1 and MSTRG.9585.1. The dysregulated lncRNAs were found on chromosome 2, 3, 7, 9, 11, 15, 19, 21, and 22, with chromosome 19 having the highest number of dysregulated transcripts. Interestingly, among the common genes that were downregulated, MSTRG.14987.25 and MSTRG.20150.1 were found across both Clinical and Healthy comparisons, suggesting that they might be consistently associated with the disease (Fig 6; Table 2).

Fifteen lncRNAs were found to be significantly dysregulated between Clinical and Subclinical groups of Sahiwal cattle (Fig 6; Table 3). Of these, ten were found to be upregulated and five were downregulated, indicating that there was a preponderance of transcriptional activation in clinically affected animals. MSTRG.6068.3 exhibited the highest upregulation (log2FC = 9.236153, p = 0.028119), followed by MSTRG.19791.1 (log2FC = 8.592562, p = 3.97E-05), which was also the most statistically significant transcript in the dataset. MSTRG.3838.3 (log2FC = 7.452015, p = 0.009358) and MSTRG.4337.4 (log2FC = 6.264562, p = 0.014789) also showed marked upregulation, while MSTRG.2975.1, MSTRG.9556.1, MSTRG.14814.1, MSTRG.1150.15, MSTRG.24249.5, and MSTRG.26275.1 demonstrated moderate upregulation. Among the downregulated transcripts, MSTRG.19363.1 showed the strongest repression (log2FC = −7.66554, p = 0.001858), followed by MSTRG.15326.1 (log2FC = −3.51961, p = 0.02543), MSTRG.20595.1 (log2FC = −2.82435, p = 0.032624), MSTRG.25350.1 (log2FC = −2.65696, p = 0.042252), and MSTRG.21967.1 (log2FC = −1.33959, p = 0.013886) (Fig 6; Table 3).

**Table 3:** Significant differentially expressed (DE) lncRNAs in Sahiwal cows with Clinical mastitis in comparison to Subclinical mastitis.

Transcript	log2FoldChange	pvalue	regulation	Length	Exon_ Count	Coding potential by CPC2	subcellular localization	Chromosome
MSTRG.1150.15	4.491404	0.04675	Up	401	2	0.014165	Nucleus	Chr1
MSTRG.2975.1	2.21538	0.027204	Up	737	2	0.0055566	Cytoplasm	Chr3
MSTRG.3838.3	7.452015	0.009358	Up	2288	6	0.0320197	Ribosome	Chr3
MSTRG.4337.4	6.264562	0.014789	Up	1198	2	0.516494	Nucleus	Chr4
MSTRG.6068.3	9.236153	0.028119	Up	2106	2	0.118962	Cytoplasm	Chr5
MSTRG.9556.1	4.820386	0.00024	Up	633	3	0.0240435	Cytoplasm	Chr8
MSTRG.14814.1	2.007176	0.001182	Up	1422	2	0.0240066	Cytoplasm	Chr14
MSTRG.19791.1	8.592562	3.97E-05	Up	1949	2	0.0160923	Nucleus	Chr19
MSTRG.24249.5	4.396851	0.004499	Up	1522	4	4.99639E-7	Nucleus	Chr26
MSTRG.26275.1	2.024821	0.0269	Up	355	2	0.107571	Cytoplasm	ChrX
MSTRG.15326.1	−3.51961	0.02543	Down	1160	2	0.0237341	Nucleus	Chr15
MSTRG.19363.1	−7.66554	0.001858	Down	854	3	0.0140057	Nucleus	Chr19
MSTRG.20595.1	−2.82435	0.032624	Down	682	2	0.0203216	Cytoplasm	Chr20
MSTRG.21967.1	−1.33959	0.013886	Down	974	2	0.161312	Cytoplasm	Chr22
MSTRG.25350.1	−2.65696	0.042252	Down	1215	2	0.0224178	Cytosol	Chr28
MSTRG.25411.1	−2.2655	0.034312	Down	521	2	0.0311598	Cytoplasm	Chr28
MSTRG.25768.1	−4.17443	0.041963	Down	466	2	0.0383082	Cytoplasm	Chr29

The transcript lengths ranged from 2288 bp (MSTRG.3838.3) to 355 bp (MSTRG.26275.1) with an exon count of two to six. Most of the transcripts in the study had two exons, as is typical of lncRNAs, with the largest transcript (MSTRG.3838.3) having six. All transcripts were confirmed as non-coding using CPC2 coding potential analysis with MSTRG.24249.5 being the lowest (most confident) score (4.99639E-7). Functional heterogeneity was observed through subcellular localization, and the cytoplasmic localization of MSTRG.6068.3, MSTRG.9556.1, MSTRG.14814.1, MSTRG.26275.1, MSTRG.20595.1, and MSTRG.21967.1 suggested miRNA sponging and ceRNA interaction functions, while the nuclear localization of MSTRG.1150.15, MSTRG.4337.4, MSTRG.19791.1, MSTRG.24249.5, MSTRG.15326.1, and MSTRG.19363.1 indicated that they were involved in transcription or epigenetic regulation. One was ribosome-associated (MSTRG.3838.3) and one was cytosol-localized (MSTRG.25350.1). The dysregulated lncRNAs were distributed across chromosomes 1, 3, 4, 5, 8, 14, 15, 19, 20, 22, 26, 28 and the X chromosome, with two lncRNAs each on chromosomes 3 and 19. Interestingly, MSTRG.6068.3 and MSTRG.24249.5 were consistently upregulated in both Clinical vs Healthy and Clinical vs Subclinical comparisons, thus suggesting that they may serve as potential markers of Clinical stage lncRNAs in Sahiwal cattle (Fig 6; Table 3).

### 3.5. Identification of known and novel LncRNAs

**Fig 7:**
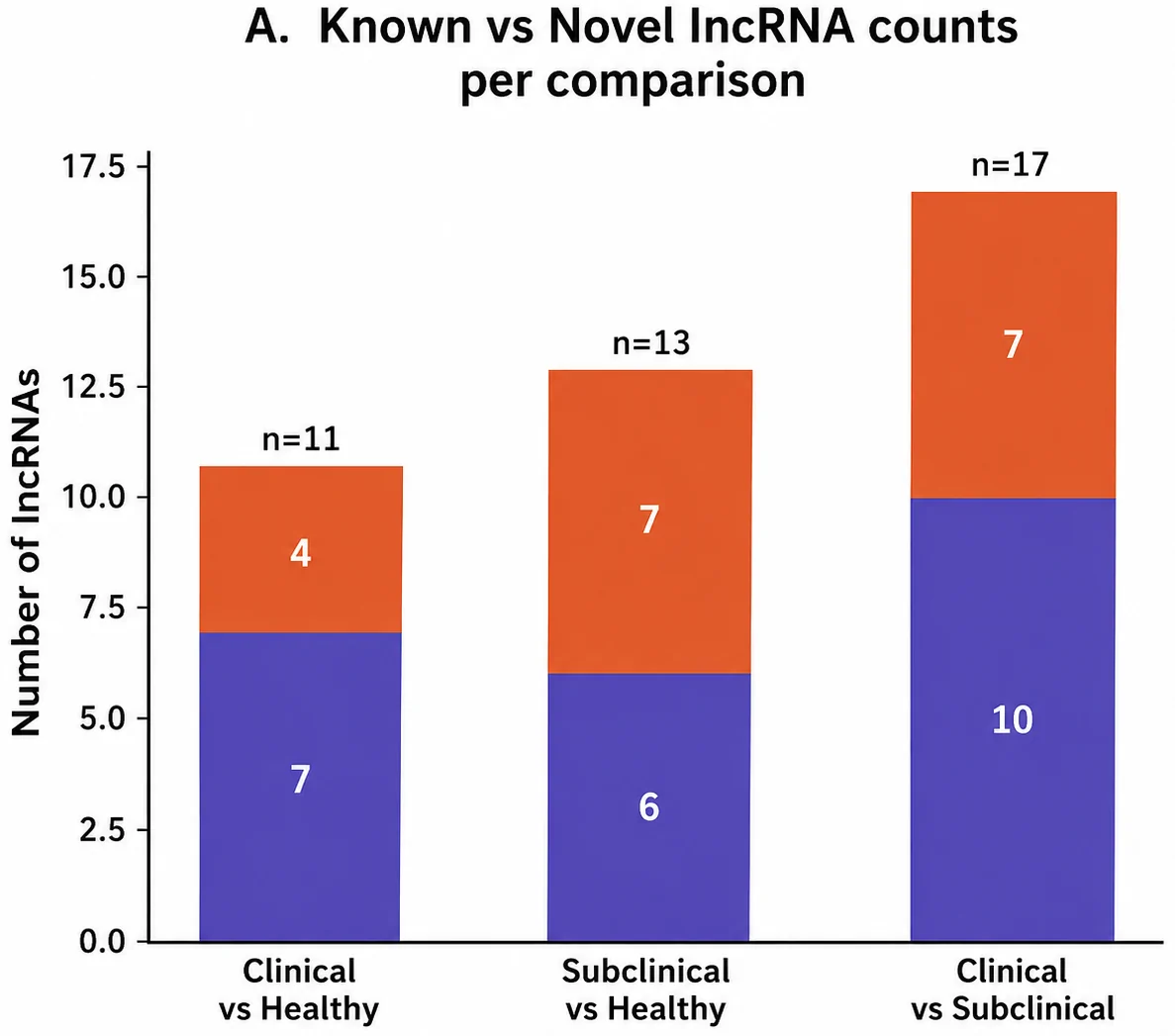
Distribution of known (purple) and new (orange) lncRNAs across Clinical vs Healthy, Subclinical vs Healthy, and Clinical vs Subclinical comparisons.

Long non-coding RNAs (lncRNAs) showing sequence similarity >80% and E-value < 1e-5 against known bovine lncRNAs were classified as known lncRNAs, while the remaining transcripts were considered novel (Fig 7). The Clinical vs Healthy comparison identified 11 dysregulated lncRNAs, including 7 known and 4 novel transcripts. Similarly, the Subclinical vs Healthy comparison detected 13 lncRNAs, comprising 6 known and 7 novel transcripts, with novel lncRNAs slightly predominating. The Clinical vs Subclinical comparison showed the highest number of dysregulated lncRNAs (17), including 10 known and 7 novel transcripts. The predominance of known lncRNAs suggests conserved regulatory roles, whereas the presence of several novel lncRNAs indicates potential unidentified regulatory mechanisms in Sahiwal cattle.

### 3.6. Integrative analysis of lncRNA-miRNA-mRNA regulatory networks

The ceRNA interaction analysis revealed several significant lncRNA-miRNA-mRNA regulatory networks associated with mastitis progression in cattle. In both Clinical vs Healthy and Subclinical vs Healthy comparisons, the upregulated lncRNA MSTRG.21615.2 showed strong interaction with bta-miR-212 and bta-miR-760-3p, targeting the genes SIRT2 and OAT, respectively. The interaction with bta-miR-212 exhibited a high interaction score (156) and favorable binding energy (−22.73 kcal/mol) with a low MFE value (−32.6), indicating stable hybridization between the lncRNA and miRNA. The target gene SIRT2, is known to be involved in NAD+ binding, along with NAD-dependent deacetylase activities. Functionally, it plays important roles in cell division and regulation of developmental processes and is localized in cellular components such as the cytoplasm, cytoskeleton, microtubules, membrane, and nucleus, indicating its involvement in cellular regulation (Table 4 and 5). Similarly, interaction with bta-miR-760-3p targeted OAT, which is found to be involved in ornithine aminotransferase activity and pyridoxal phosphate binding. It participates in arginine catabolism and proline biosynthesis and is localized in the mitochondrion, suggesting a role in amino acid metabolism and energy regulation. The negative binding energies and significant p-values (<0.05) support the reliability of these predicted ceRNA interactions.

**Table 4:** Predicted lncRNA-miRNA-mRNA interactions associated with Sahiwal cows with Clinical mastitis in comparison to healthy cows by miRanda and RNAhybrid.

Comparison	Regulation	Target lncRNA	Target Length	miRNA	miRNA Length	Score	Energy (kcal/mol)	MFE	P-value	Target Gene
Clinical vs Healthy	Up	MSTRG.21615.2	917	bta-miR-212	23	156	−22.73	−32.6	0.024	SIRT2
Clinical vs Healthy				bta-miR-760-3p	20	154	−26.52	−32.2	0.03	OAT
Subclinical vs Healthy	Up	MSTRG.21615.2	917	bta-miR-212	23	156	−22.73	−32.6	0.02453	SIRT2
Subclinical vs Healthy				bta-miR-760-3p	20	154	−26.52	−32.2	0.030205	OAT
Clinical vs Subclinical	Down	MSTRG.25768.1	466	bta-miR-2425-5p	22	154	−28.52	−31.3	0.018447	ADAMTS1
Clinical vs Subclinical	Down	MSTRG.19363.1	854	bta-miR-1296	22	156	−33.87	−37.9	0.002646	KPNA7
Clinical vs Subclinical	up	MSTRG.19791.1	1948	bta-miR-2382-5p	22	150	−32	−38.1	0.01018	SOD3
Clinical vs Subclinical	up	MSTRG.1150.15	401	bta-miR-92b	22	163	−26.34	−32.4	0.009212	PTEN

**Table 5:** Functional annotation of predicted target genes in the lncRNA-miRNA-mRNA ceRNA network associated with mastitis in Sahiwal cattle.

miRNA	Target Gene	Molecular Function (MF)	Biological Process (BP)	Cellular Component (CC)
bta-miR-212	SIRT2	Metal ion binding, NAD+ binding, NAD-dependent protein demyristoylase activity, NAD-dependent protein depalmitoylase activity, NAD-dependent protein lysine deacetylase activity, and tubulin deacetylase activity.	Cell division, negative regulation of developmental process, and regulation of multicellular organismal process	Cell membrane, cell projection, chromosome, cytoplasm, cytoskeleton, membrane, microtubule, and nucleus.
bta-miR-760-3p	OAT	Identical protein binding, ornithine aminotransferase activity, and pyridoxal phosphate binding.	L-arginine catabolic process to L-glutamate, L-arginine catabolic process to proline via ornithine, and L-proline biosynthetic process	Mitochondrion
bta-miR-2425-5p	ADAMTS1	Heparin binding, metalloendopeptidase activity, and zinc ion binding.	Extracellular matrix organization, heart trabecula formation, kidney development, negative regulation of angiogenesis, ovulation from ovarian follicle, positive regulation of G1/S transition of mitotic cell cycle, positive regulation of vascular associated smooth muscle cell migration, positive regulation of vascular associated smooth muscle cell proliferation, and proteolysis.	Extracellular matrix, Secreted
bta-miR-1296	KPNA7	Nuclear import signal receptor activity and nuclear localization sequence binding.	NLS-bearing protein import into nucleus.	Nucleus
bta-miR-2382-5p	SOD3	Copper ion binding and superoxide dismutase activity.	Response to hypoxia.	Golgi apparatus, Membrane, nucleus, secreted
bta-miR-92b	PTEN	Enzyme binding, phosphatidylinositol-3,4,5-trisphosphate 3-phosphatase activity, protein serine/threonine phosphatase activity, and protein tyrosine phosphatase activity.	Apoptotic process, cell motility, negative regulation of cell communication, negative regulation of cell population proliferation, negative regulation of cellular component organization, negative regulation of developmental process, negative regulation of multicellular organismal process, negative regulation of signaling, nervous system development, phosphatidylinositol 3-kinase/protein kinase B signal transduction, phosphatidylinositol dephosphorylation, positive regulation of macromolecule metabolic process, regulation of cell cycle, regulation of cell differentiation, and regulation of phosphatidylinositol 3-kinase/protein kinase B signal transduction.	Cell projection, cytoplasm,

In the Clinical vs Subclinical comparison, multiple distinct regulatory interactions were identified, indicating molecular differences between severe and moderate stages of infection. The downregulated lncRNA MSTRG.25768.1 interacted with bta-miR-2425-5p to regulate ADAMTS1, with a strong interaction score (154) and stable MFE (−31.3). ADAMTS1 is involved in extracellular matrix organization, angiogenesis regulation, cell proliferation, and proteolysis and is primarily localized in the extracellular matrix as a secreted protein, suggesting reduced regulation of tissue remodeling pathways during progression from subclinical to clinical mastitis (Table 4 and 5). Another downregulated lncRNA, MSTRG.19363.1, targeted KPNA7 through bta-miR-1296 and showed the strongest binding stability among all interactions, with an energy value of −33.87 kcal/mol and MFE of −37.9. The low p-value (0.002646) indicates a highly significant interaction. KPNA7 is associated with nuclear transport and gene regulatory mechanisms, implying possible alterations in cellular signaling and transcriptional regulation during disease progression. Among the upregulated interactions in Clinical vs Subclinical animals, MSTRG.19791.1 interacted with bta-miR-2382-5p targeting SOD3. The strong binding energy (−32 kcal/mol) and low MFE (−38.1) indicate highly stable interaction. SOD3 encodes extracellular superoxide dismutase, an important antioxidant enzyme that protects tissues from oxidative stress generated during inflammation. Upregulation of this axis may therefore represent a compensatory antioxidant defense mechanism in mastitic animals. Similarly, the upregulated lncRNA MSTRG.1150.15 interacted with bta-miR-92b targeting PTEN, a critical regulator of the PI3K/AKT signaling pathway involved in apoptosis, immune response, and cell survival. The significant interaction score (163), favorable energy (−26.34 kcal/mol), and low p-value (0.009212) suggest that this ceRNA network may play an important role in controlling inflammatory signaling and immune cell activity during mastitis (Table 4,5).

**Fig 8:**
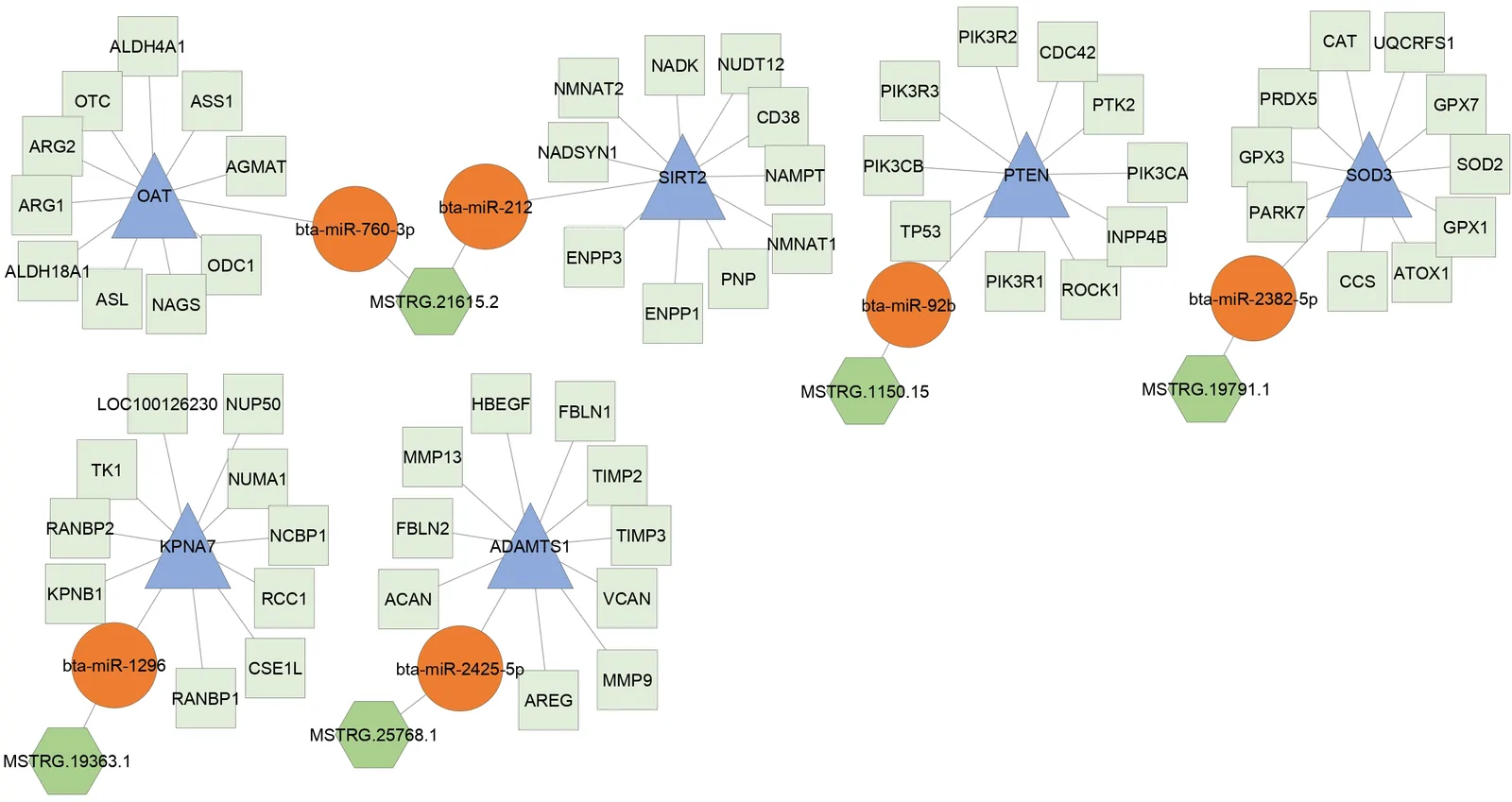
CeRNA and PPI Network in Clinical vs Healthy, Subclinical vs Healthy, and Clinical vs Subclinical comparisons. The network illustrates five independent regulatory axes connecting long non-coding RNAs (lncRNAs, green diamonds), microRNAs (miRNAs, orange-red circle), target genes (steel-blue star/burst nodes), and their PPI partner proteins (yellow-green rectangles). Edges represent lncRNA-miRNA sponging interactions, miRNA-mediated target gene suppression, and PPI associations derived from STRING database confidence scores.

The network defines five modules of ceRNAs that are active in the Clinical vs Healthy comparison, Subclinical vs Healthy comparison, and Clinical vs Subclinical comparison (Fig 8). The first module is a Dual-hub network, where lncRNA MSTRG.21615.2 is a shared ceRNA that relieves miRNA-mediated suppression of two functionally related hub genes, namely OAT and SIRT2. Interacting partners of the OAT include ARG1, ARG2, ASS1, OTC, NAGS, AGMAT, ASL, ODC1, and ALDH4A1/ALDH18A1, whereas SIRT2 partners include NAMPT, NMNAT1, NMNAT2, NADSYN1, NADK, NUDT12, CD38, ENPP1, ENPP3, and PNP. The second network involves the critical tumour suppressor PTEN which is controlled by sponging bta-miR-92b through MSTRG.1150.15. PTEN interacts with PI3K family members (PIK3CA, PIK3CB, PIK3R1, PIK3R2, PIK3R3), TP53, CDC42, PTK2, INPP4B and ROCK1, all of which signify activation of the PI3K/AKT signalling axis and cytoskeletal remodelling. The third network comprises an extracellular antioxidant enzyme called SOD3, which is regulated by MSTRG.19791.1 sponging bta-miR-2382-5p and many of its PPI partners are involved in oxidative stress and imbalance of reactive oxygen species, such as CAT, GPX1, GPX3, GPX7, UQCRFS1, PRDX5, PARK7, SOD2, CCS, and ATOX1 (Fig 8). The fourth one involves the nuclear import adaptor KPNA7, regulated by MSTRG.19363.1 sponging bta-miR-1296, and associated with partners RANBP2, RANBP1, KPNB1, NUP50, NUMA1, NCBP1, RCC1, LOC100126230, CSE1L and TK1, which include perturbations of nucleocytoplasmic transport and of regulation of mitosis. The fifth network consists of secreted metalloprotease ADAMTS1 which is regulated by MSTRG.25768.1 sponging bta-miR-2425-5p, and whose PPI partners include MMP9, MMP13, TIMP2, TIMP3, VCAN, ACAN, FBLN2, HBEGF, and AREG, all related to the dysregulation of extracellular matrix remodelling and growth factor signalling.

### 3.7. Structural Interaction between lncRNA-miRNA

The results of the six predicted lncRNA-miRNA interaction pairs showed that all docking runs generated 9–14 clusters for each complex. The highest clusters had Z-scores between −1.2 and −2.2, indicating that they are statistically distinct from the rest of the cluster ensemble. The complex between MSTRG.1150.15 and bta-miR-92b exhibited the best predicted interaction with the lowest HADDOCK score (−15.4 ± 6.9 kcal/mol) and the most negative Z-score (−2.2). This complex also exhibited a very low RMSD (1.4 ± 0.9 Å), a buried surface area (BSA) of 1390.1 ± 105.0 Å² and very favourable van der Waals (−47.6 ± 7.2 kcal/mol) and electrostatic (−51.3 ± 18.9 kcal/mol) interaction energies, which suggest a stable and well-converged docking solution. The second best-scoring interaction was the MSTRG.25768.1-bta-miR-2425-5p complex, with a HADDOCK score of −10.8 ± 7.3 kcal/mol and a Z score of −1.9. Specifically, it had the lowest RMSD (0.2 ± 0.2 Å), excellent structural convergence, along with the most favourable electrostatic energy (−78.8 ± 9.6 kcal/mol), and BSA (1514.2 ± 76.5 Å²), which indicated highly stable, electrostatic interaction. The interaction between MSTRG.21615.2 and bta-miR-760-3p also showed favorable binding characteristics, with a HADDOCK score of −6.4 ± 6.9 kcal/mol, a Z-score of −1.9, and a low RMSD (1.1 ± 0.8 Å). This complex had the largest buried surface area (1845.2 ± 172.8 Å²) of all docked pairs, with weaker electrostatic interactions (−14.8 ± 5.1 kcal/mol). The MSTRG.21615.2-bta-miR-212 complex, on the other hand, showed the most unfavourable interaction with the highest RMSD (11.0 ± 0.2 Å), the lowest Z-score (−1.2), and the only positive HADDOCK score (+15.5 ± 3.7 kcal/mol). In addition, the positive electrostatic energy (+59.2 ± 45.6 kcal/mol) suggested electrostatic repulsion even though the BSA of the complex is relatively large (1574.6 ± 137.7 Å²). Intermediate docking performance was observed in the other two complexes. MSTRG.19363.1-bta-miR-1296 yielded a HADDOCK score of −7.8 ± 4.2 kcal/mol with a Z-score of −1.6, RMSD of 9.1 ± 0.2 Å, and BSA of 1223.2 ± 26.2 Å², supported by favorable van der Waals (−44.4 ± 2.8 kcal/mol) and electrostatic (−27.2 ± 11.8 kcal/mol) interactions. Similarly, MSTRG.19791.1–bta-miR-2382-5p exhibited a HADDOCK score of −0.6 ± 1.7 kcal/mol, a Z-score of −1.8, RMSD of 6.7 ± 0.4 Å, and BSA of 1188.8 ± 74.8 Å², with negative van der Waals (−40.9 ± 5.3 kcal/mol) and electrostatic (−25.8 ± 9.4 kcal/mol) energies, indicative of moderately stable yet structurally stable binding interactions (Fig 9; Table 6).

**Table 6:** HADDOCK docking results showing top-ranked clusters based on HADDOCK score (kcal/mol).

#	Target lncRNA	miRNA	No. of clusters	Top cluster	Cluster size	HADDOCK score	Z-score	RMSD (from min)	vdW energy (kcal/mol)	Electrostatic (kcal/mol)	Desolvation (kcal/mol)	Restraints violation	BSA (A²)
1	MSTRG.21615.2	bta-miR-212	9	Cluster 3	12	+15.5 ± 3.7	−1.2	11.0 ± 0.2	−58.6 ± 9.2	+59.2 ± 45.6	22.3 ± 1.7	399.4 ± 59.7	1574.6 ± 137.7
2	MSTRG.21615.2	bta-miR-760-3p	13	Cluster 8	7	−6.4 ± 6.9	−1.9	1.1 ± 0.8	−49.5 ± 6.8	−14.8 ± 5.1	36.5 ± 2.6	95.1 ± 32.2	1845.2 ± 172.8
3	MSTRG.25768.1	bta-miR-2425-5p	12	Cluster 1	22	−10.8 ± 7.3	−1.9	0.2 ± 0.2	−48.1 ± 3.1	−78.8 ± 9.6	22.3 ± 1.6	308.1 ± 78.9	1514.2 ± 76.5
4	MSTRG.19363.1	bta-miR-1296	10	Cluster 3	13	−7.8 ± 4.2	−1.6	9.1 ± 0.2	−44.4 ± 2.8	−27.2 ± 11.8	19.3 ± 0.4	227.3 ± 40.6	1223.2 ± 26.2
5	MSTRG.19791.1	bta-miR-2382-5p	9	Cluster 2	19	−0.6 ± 1.7	−1.8	6.7 ± 0.4	−40.9 ± 5.3	−25.8 ± 9.4	22.2 ± 1.2	233.0 ± 24.7	1188.8 ± 74.8
6	MSTRG.1150.15	bta-miR-92b	14	Cluster 2	12	−15.4 ± 6.9	−2.2	1.4 ± 0.9	−47.6 ± 7.2	−51.3 ± 18.9	22.1 ± 1.8	204.9 ± 76.6	1390.1 ± 105.0

**Fig 9:**
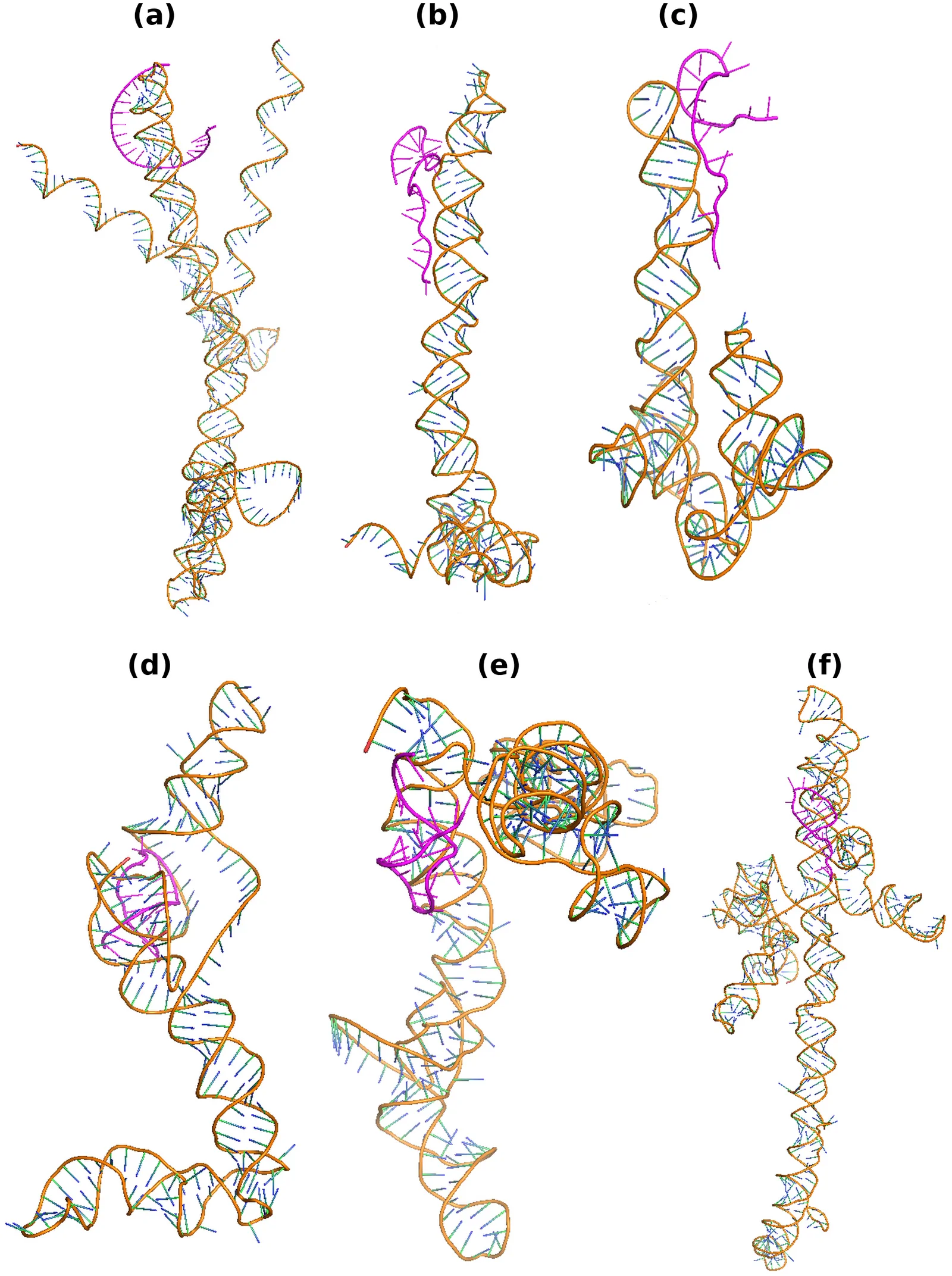
Three-dimensional structural interaction between lncRNAs and miRNAs. Predicted secondary structures of six lncRNA-miRNA interaction complexes (a-f) generated using RNA structure visualization software. The orange backbone represents the lncRNA molecule, while the pink region highlights the bound miRNA. (a) MSTRG.1150.15- bta-miR-92b (b) MSTRG.19791.1- bta-miR-2382-5p (c) MSTRG.19363.1- bta-miR-1296 (d) MSTRG.21615.2-bta-miR-212 (e)MSTRG.21615.2- bta-miR-760-3p (f) MSTRG.25768.1- bta-miR-2425-5p.

### 3.8. Molecular dynamics simulations analysis

**Fig 10:**
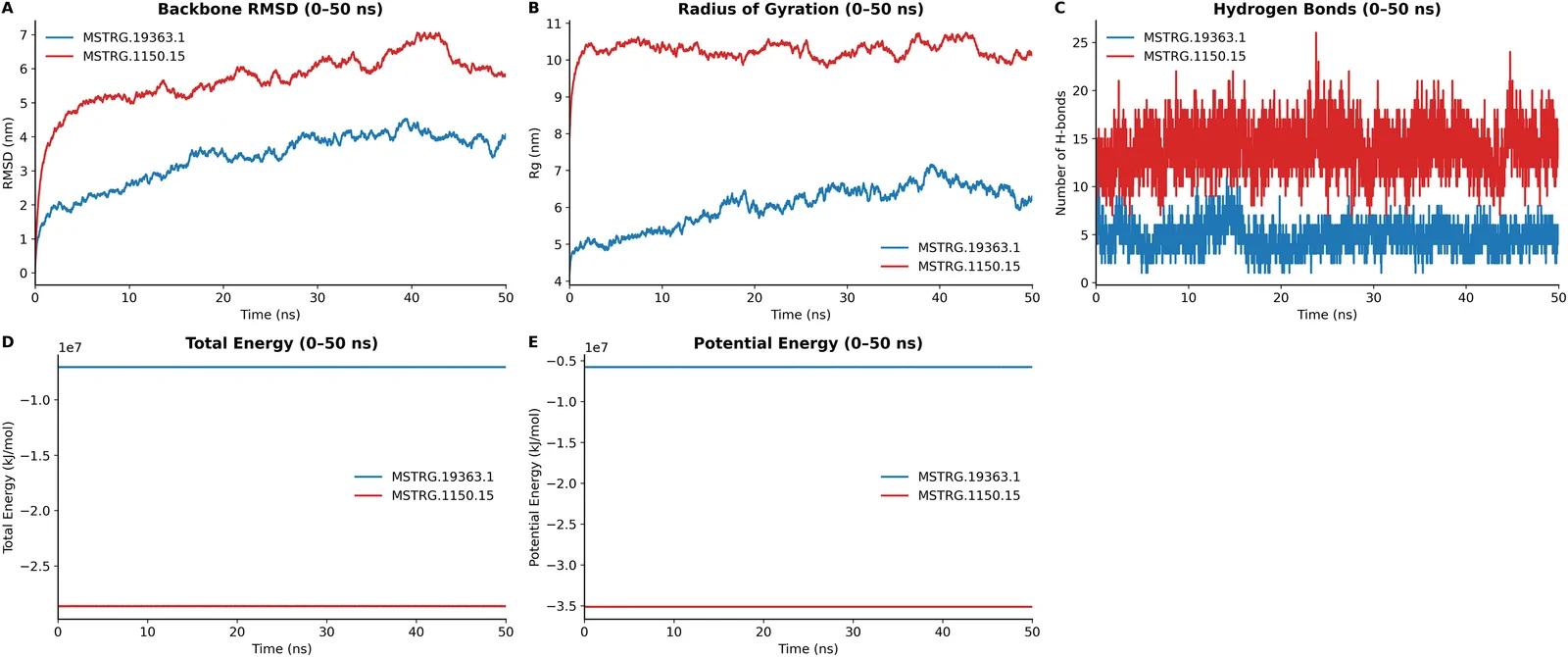
The MD simulation of the MSTRG. **1150.15-bta-miR-92b and MSTRG.19363.1** with bta-miR-1296 complex (A) RMSD of the RNA complex plotted against simulation time (B) Radius of Gyration (Rg) plot (C) No. of Hydrogen Bond (D) Total energy and (E) Potential energy.

Owing to computational resource constraints, molecular dynamics (MD) simulations were performed only for two selected lncRNA-miRNA complexes. The MSTRG.1150.15-bta-miR-92b interaction was selected based on its significant differential expression (p = 0.009212), favorable HADDOCK docking score (−15.4 ± 6.9 kcal/mol), large buried surface area (1390.1Å²), high miRanda score (163), strong miRanda binding energy (−26.34 kcal/mol), and low minimum free energy (MFE; −32.4 kcal/mol). The MSTRG.19363.1-bta-miR-1296 complex was selected based on its superior statistical significance (p = 0.002646), favorable HADDOCK docking score (−7.8 ± 4.2 kcal/mol), large buried surface area (1223.2 Å²), strong miRanda binding energy (33.87 kcal/mol), high miRanda score (156), low MFE (37.9 kcal/mol), and its involvement in the biologically relevant comparison group, Clinical vs Subclinical mastitis. The two lncRNA-miRNA docked complexes show different stability profiles under 50 ns MD simulation. The RMSD trajectory of the MSTRG.19363.1-miRNA complex was comparatively low, around ~3.9 nm, and slowly converged to a stable conformation (Fig 10A), while the RMSD trajectory of the MSTRG.1150.15-miRNA complex was higher and more rapidly diverged from the initial docked conformation, reaching ~5.8 nm by the end of the trajectory, suggesting more conformational drift from the initial docked conformation, and hence a less stably converged structure on a simulated timescale. The same trend was observed in the radius of gyration where MSTRG.19363.1 was relatively small (Rg ≈ 6.5 nm), whereas MSTRG.1150.15 was significantly larger and more expanded (Rg ≈ 10.1 nm), indicating a more compact structure for MSTRG.19363.1 and a more extended conformation or partial unfolding of the for MSTRG.1150.15 complex under the simulation conditions (Fig 10B). The RMSD and Rg trends suggest that the MSTRG.19363.1 complex with its targeted miRNA is more conformationally stable and compact.

The opposite trend was observed in the number of H-bonds where MSTRG.1150.15 had a higher number of H-bonds (~15–16 direct bonds, up to ~30 pairs within 0.35 nm) than MSTRG.19363.1 (~4–6 H-bonds) (Fig 10c). It does not necessarily mean that the MSTRG.1150.15 complex is more tightly bound, but rather, that it samples the conformational space more freely, so that more transient contacts can be formed and broken which explains its higher RMSD/Rg. By contrast, the smaller, more stable H-bond network in MSTRG.19363.1 is maintained in a structurally rigid, low-RMSD complex, indicating that the interactions exhibited are more persistent in terms of function than more numerous. The total and potential energy profiles of both complexes were constant throughout the trajectory, and showed no significant drift (Fig 10d, 10e), suggesting that both systems reached energetic equilibration under the simulation conditions. The potential energy of both complexes was tracked for the entire trajectory of 50 ns to assess the stability and optimization of the docked complexes during the simulation. For MSTRG.19363.1-bta-miR-1296, the potential energy changed from −7,059,294.0 kJ/mol at 0 ns to −7,058,631.0 kJ/mol at 50 ns, while for MSTRG.1150.15-bta-miR-92b, the potential energy changed from −35,185,640.0 kJ/mol at 0 ns to −35,138,224.0 kJ/mol at 50 ns. In both cases, the potential energy was stable during the simulation, and with very little net drift, so both complexes were able to reach energetic equilibration under the simulated conditions. The two most direct structural-stability measurements, RMSD and Rg, also support MSTRG.19363.1 as the more conformationally stable lncRNA-miRNA complex, as it was selected as the representative stable complex.

## 4. Discussion

The present study provides a comprehensive overview of the ceRNA regulatory landscape associated with mastitis progression in Sahiwal cows. Analysis of milk somatic cells across the Clinical vs Healthy, Subclinical vs Healthy, and Clinical vs Subclinical comparisons identified five key lncRNA-miRNA-mRNA regulatory axes that may contribute to the molecular mechanisms underlying the transition from health to subclinical and clinical mastitis.

In the Clinical vs Healthy and Subclinical vs Healthy comparisons, MSTRG.21615.2 was predicted to interact with bta-miR-212 and bta-miR-760-3p, thereby regulating SIRT2 and OAT, respectively. SIRT2 is a NAD + -dependent deacetylase involved in energy metabolism, cellular survival, and redox homeostasis. The SIRT2-centered protein-protein interaction network, comprising NAMPT, NMNAT1, NMNAT2, CD38, and NADSYN1, suggests that perturbation of NAD+ metabolism may be associated with mastitis development. Previous studies have demonstrated that miR-212 regulates lipogenesis in bovine mammary epithelial cells by targeting SIRT2 [36]. Therefore, dysregulation of the MSTRG.21615.2-bta-miR-212-SIRT2 axis may influence both metabolic homeostasis and mammary gland function during mastitis. Likewise, OAT plays an important role in amino acid metabolism and cellular energy balance. Derepression of OAT through the MSTRG.21615.2-bta-miR-760-3p interaction may represent a metabolic adaptation to inflammatory stress in the mammary gland.

The Clinical vs Subclinical comparison among the upregulated lncRNAs, MSTRG.19791.1 was predicted to sponge bta-miR-2382-5p, thereby relieving repression of SOD3, a key extracellular antioxidant enzyme. The SOD3-centered network, including CAT, GPX1, PRDX5, and ATOX1, defines an oxidative stress defense module that may protect mammary tissue against excessive reactive oxygen species generated during inflammation. Previous studies have shown that bta-miR-2382-5p negatively regulates SOD3 in bovine mammary epithelial cells [37]. Thus, activation of the MSTRG.19791.1-bta-miR-2382-5p-SOD3 axis may represent a compensatory mechanism aimed at limiting oxidative damage during mastitis.

Similarly, MSTRG.1150.15 was predicted to sequester bta-miR-92b, resulting in increased PTEN expression. PTEN is a well-established negative regulator of the PI3K/AKT signaling pathway and plays a crucial role in controlling cell survival, proliferation, and inflammatory responses. Previous studies reported that miR-92b exerts anti-inflammatory effects by targeting PTEN, activating the PI3K/AKT/β-catenin pathway, and suppressing TLR4/NF-κB-mediated inflammation [38]. Consequently, dysregulation of the MSTRG.1150.15-bta-miR-92b-PTEN axis may contribute to enhanced inflammatory signaling, apoptosis, and tissue injury during mastitis progression.

In contrast, two lncRNAs were downregulated in the Clinical vs Subclinical comparison, indicating suppression of specific biological processes in severe disease. Downregulation of MSTRG.19363.1 may reduce its sponging activity toward bta-miR-1296, thereby enhancing repression of KPNA7, a member of the karyopherin family involved in nucleocytoplasmic transport. The associated interaction network, involving RAN-binding proteins, NUP50, and CSE1L, suggests impairment of nuclear transport and cell-cycle regulation in severely affected mammary tissue, potentially limiting epithelial regeneration and tissue repair. Likewise, MSTRG.25768.1 was predicted to interact with bta-miR-2425-5p and regulate ADAMTS1. Previous studies have demonstrated that bta-miR-2425-5p suppresses ADAMTS1 expression and inhibits triglyceride synthesis in bovine mammary epithelial cells [39]. Therefore, reduced expression of MSTRG.25768.1 may enhance miR-2425-5p-mediated repression of ADAMTS1, potentially contributing to altered mammary gland metabolism and functional impairment during mastitis.

The lncRNA-miRNA-mRNA interaction network predicted in the present study is in line with previous studies emphasizing the role of the lncRNA-associated regulatory network in bovine mastitis. For instance, the lncCRHR1-miR-302d-FGF19 axis has been reported to regulate the proliferation, apoptosis, and inflammatory responses in the bovine mammary epithelial cells (BMEC) through ceRNA mechanism [40]. In the same manner, lncRNA TCONS_00058979 has been demonstrated to be a promoter of inflammatory injury in bovine mammary epithelial cells, while its suppression helps to reduce inflammation and cellular damage to the cells [41]. The findings of this study support the possible biological relevance of the lncRNA-miRNA-mRNA interactions predicted in this study. Together, these findings reinforce the emerging role of lncRNAs as critical regulators of mammary gland immunity, metabolism, and cellular homeostasis. Altogether, the identified lncRNA-miRNA-mRNA axes suggest that mastitis progression in Sahiwal cows is associated with coordinated dysregulation of metabolic, oxidative stress, inflammatory, apoptotic, and cellular transport pathways. These ceRNA networks provide new insights into the molecular mechanisms underlying mastitis and may serve as potential biomarkers and therapeutic targets for improving mastitis resistance in dairy cattle.

## 5. Conclusion

This study provides a detailed lncRNA-miRNA-mRNA network analysis to identify molecular regulatory pathways involved in the progression of mastitis in Sahiwal cows. Transcriptome-wide screening identified 757 high-confidence lncRNAs of which 41 were differentially expressed between the Clinical vs Healthy, Subclinical vs Healthy and Clinical vs Subclinical comparison (23 known and 18 novel) and differentially expressed patterns suggested stage-specific regulation of transcription. There were fewer lncRNAs that were dysregulated at subclinical mastitis than were dysregulated at clinical mastitis, which indicates that as the disease worsens there may be more cellular stress and inflammation in clinical mastitis. The integrative ceRNA-PPI network identified a relationship between lncRNAs and immune-, oxidative-, transport-, and matrix-remodelling-related pathways via the regulating modulation of key miRNAs and their target genes. Of particular note, oxidative stress regulation was found as a key characteristic of mastitis, with lncRNAs interacting with miRNAs that regulate SOD3 and OAT. The conformational stability of MSTRG.19363.1-bta-miR-1296 complex was validated by structural modeling and molecular dynamics simulation, suggesting it could be involved in regulating nucleocytoplasmic transport in disease progression. All of these results give new insights in the molecular mechanisms involved in the progression of mastitis, and highlight potential regulatory biomarker for early diagnosis and therapeutic targeting. These lncRNA-mediated networks in bovine health and productivity will be further validated in experimental models of bovine mammary epithelial cells and in functional assays that will evaluate the biological significance of these networks on a large scale.

### 5.1. Limitations

A major limitation of this study is the small number of animals included in each group (n = 3), which may have reduced the statistical power of the differential-expression and ceRNA network analyses. In addition, statistical significance was assessed using a nominal p-value threshold of <0.05 without correction for multiple testing. Consequently, some of the identified differentially expressed lncRNAs and predicted regulatory interactions may represent false-positive findings. The results should therefore be interpreted cautiously and validated in larger, independent sample sets using multiple-testing correction and experimental assays.

## Supporting information

S1 File Predicted interacting nucleotide residues of lncRNA-miRNA complexes used for molecular docking analysis. (DOCX)
