# Supplementary material for: Identification of differentially expressed lncRNAs and candidate ceRNA networks across Mastitis stages in Sahiwal cattle
Source: PLoS One. 2026 Sep 15;21(9):e0357796. doi: 10.1371/journal.pone.0357796 (PMC13577530; doi:10.1371/journal.pone.0357796)
Supplement: Supplementary file 1 [file pone.0357796.s001.docx]

**Table S1:** Predicted interacting nucleotide residues of lncRNA-miRNA complexes used for molecular docking analysis.

|  | **Target lncRNA** | **miRNA** | **lncRNA interacting residue** | **miRNA interacting residue** |
| --- | --- | --- | --- | --- |
| 1 | MSTRG.21615.2 | bta-miR-212 | 277-298 | 2-15 |
| 2 | MSTRG.21615.2 | bta-miR-760-3p | 704-723 | 2-19 |
| 3 | MSTRG.25768.1 | bta-miR-2425-5p | 286-308 | 2-18 |
| 4 | MSTRG.19363.1 | bta-miR-1296 | 31-52 | 2-21 |
| 5 | MSTRG.19791.1 | bta-miR-2382-5p | 791-816 | 2-21 |
| 6 | MSTRG.1150.15 | bta-miR-92b | 137-158 | 2-20 |
